# Retrospective analysis of factors associated with outcome in veno-venous extra-corporeal membrane oxygenation

**DOI:** 10.1186/s12890-023-02591-5

**Published:** 2023-08-16

**Authors:** Thomas Orthmann, Zied Ltaief, Jean Bonnemain, Matthias Kirsch, Lise Piquilloud, Lucas Liaudet

**Affiliations:** 1 The Department of Adult Intensive Care Medicine, University Hospital Medical Center, Lausanne, 1011 Switzerland; 2 The Faculty of Biology and Medicine, University of Lausanne, Lausanne, 1011 Switzerland; 3 The Department of Cardiac Surgery, University Hospital Medical Center, Lausanne, 1011 Switzerland

**Keywords:** Veno-venous extracorporeal membrane oxygenation, Acute respiratory failure, Acute respiratory distress syndrome, Mechanical ventilation, Driving pressure

## Abstract

**Background:**

The outcome of Veno-Venous Extracorporeal Membrane Oxygenation (VV-ECMO) in acute respiratory failure may be influenced by patient-related factors, center expertise and modalities of mechanical ventilation (MV) during ECMO. We determined, in a medium-size ECMO center in Switzerland, possible factors associated with mortality during VV-ECMO for acute respiratory failure of various etiologies.

**Methods:**

We retrospectively analyzed all patients treated with VV-ECMO in our University Hospital from 2012 to 2019 (pre-COVID era). Demographic variables, severity scores, MV duration before ECMO, pre and on-ECMO arterial blood gases and respiratory variables were collected. The primary outcome was ICU mortality. Data were compared between survivors and non-survivors, and factors associated with mortality were assessed in univariate and multivariate analyses.

**Results:**

Fifty-one patients (33 ARDS, 18 non-ARDS) were included. ICU survival was 49% (ARDS, 39%; non-ARDS 67%). In univariate analyses, a higher driving pressure (DP) at 24h and 48h on ECMO (whole population), longer MV duration before ECMO and higher DP at 24h on ECMO (ARDS patients), were associated with mortality. In multivariate analyses, ECMO indication, higher DP at 24h on ECMO and, in ARDS, longer MV duration before ECMO, were independently associated with mortality.

**Conclusions:**

DP on ECMO and longer MV duration before ECMO (in ARDS) are major, and potentially modifiable, factors influencing outcome during VV-ECMO.

**Supplementary Information:**

The online version contains supplementary material available at 10.1186/s12890-023-02591-5.

## Introduction

Veno-venous extra-corporeal membrane oxygenation (VV-ECMO) is increasingly used as a therapeutic option in patients with respiratory failure refractory to conventional management, as attested by the latest ELSO registry reporting more than 30,000 VV-ECMO runs over the past 5 years (https://www.elso.org/registry/elsoliveregistrydashboard.aspx). The acute respiratory distress syndrome (ARDS) is the most common indication for VV-ECMO [1], generally started on the basis of severe hypoxemia or respiratory acidosis unresponsive to optimization of mechanical ventilation and the use of rescue therapies (inhaled nitric oxide and prone positioning) [2, 3]. Other less frequent indications for VV-ECMO are severe acute asthma, bridge to lung transplantation (LTx) and severe primary graft dysfunction (PGD) after LTx, as well as refractory bronchopleural fistula [3].

Several retrospective studies reported VV-ECMO mortality from 30 to 70% in ARDS (pre-COVID era) [4–6] and from 5 to 10% in asthma [7, 8]. In addition, it was previously reported that about 60% of patients receiving ECMO for bridge to LTx could be successfully transplanted, with survival of about 90% in these patients [9, 10]. More recently, cohort studies in COVID-19 ARDS reported VV-ECMO mortality rates of ~ 40–60% [11, 12]. Two landmark clinical trials compared VV-ECMO to conventional treatment in ARDS, the CESAR [13] and the EOLIA [14] trials. Although these studies reported non-significant reduction of mortality with VV-ECMO (35% vs. 46%, *p* = 0.09, CESAR, 37% vs. 45%, *p* = 0.07, EOLIA), a post-hoc Bayesian analysis of the EOLIA trial suggested a potential survival benefit of VV-ECMO [15], and a recent individual patient data meta-analysis of these two RCTs indicated a significant decrease of 90-day mortality with VV-ECMO in ARDS [16]. Also, in COVID-19, several emulated target trials using observational data revealed a better outcome with VV-ECMO than with conventional ventilation in patients with severe ARDS [17, 18].

Many factors may influence the chances of survival in patients under VV-ECMO, including disease severity, advanced age, the delay to ECMO initiation and the center expertise and ECMO volume [19]. Furthermore, outcome may be influenced by the applied ventilatory strategy during ECMO support [20]. Presently, there are no formal guidelines regarding such strategy, but the consensus is to use low volume and low pressure ventilation in order to prevent further ventilator-induced lung injury (VILI) [21]. Whether a strategy of ultraprotective ventilation, using very low volumes and pressures, should be used in all or in a subset of ECMO patients, is currently debated [22].

In the present retrospective cohort study, we intended to assess the outcome of VV-ECMO (ICU survival) regardless of its indication, to evaluate the strategies applied of mechanical ventilation during ECMO support and to determine possible factors associated with mortality. For this purpose, we retrospectively analyzed all VV-ECMO treatments performed in both ARDS and non-ARDS patients over a 7-year period prior to the COVID-19 pandemics in our medium volume center in Switzerland (40–50 ECMO treatments annually, including 8–10 VV-ECMO).

## Materials and methods

### Study setting and participants

The study was approved by the local ethics committee (Commission Cantonale d’Ethique de la Recherche sur l’Etre Humain, CER-VD), as a retrospective analysis of clinical and biological variables with waiver of consent, with the exclusion of patients who specified their refusal to have their data used for research purposes (Nr: 2017 − 01184). All patients treated with VV-ECMO for various indications from January 2012 to May 2019 in our tertiary multidisciplinary ICU were included. Indications for VV-ECMO were determined by the physicians in charge of the patient, primarily based on severe hypoxemia and/or severe non-treatable hypercapnia.

### VV-ECMO management

ECMO cannulas were inserted by a cardiac surgeon, using a femoro-jugular approach in most patients. Initial ECMO settings (Maquet Cardiohelp ECLS system®) comprised a sweep gas flow adapted to maintain a PaCO_2_ of 35–45 mm Hg, a sweep gas fraction of oxygen (F_S_O_2_) of 100% and a pump flow of 40–60 ml/kg. Systemic anticoagulation with non-fractionated heparin was adapted to achieve an Activated Coagulation Time (ACT) of 180–220 s or an anti-Xa activity of 0.25–0.4. Sedation was maintained with Propofol (2–4 mg/kg/h) or Midazolam (0.05–0.15 mg/kg/h). Indications for muscle paralysis were at the discretion of the physicians in charge and was achieved with rocuronium (0.6 mg/kg, repeated bolus) or cisatracurium (0.15 mg/kg, bolus, 60–120 µg/kg/h, continuous infusion). Criteria for VV-ECMO weaning were arterial O_2_ saturation ≥ 88% with FiO_2_ < 0.6 and PaCO_2_ < 55 mm Hg at a plateau pressure (Pplat) < 30 cm H_2_O and a respiratory rate (RR) < 30, under zero sweep gas flow for at least two hours of weaning test (adapted from [23]).

### Mechanical ventilation during VV-ECMO

Mechanical ventilation after the insertion of VV-ECMO was performed in volume or pressure-controlled mode, targeting a tidal volume (Vt) < 6mL/kg predicted body weight, a RR of 10–15/min and a positive end-expiratory pressure (PEEP) of 5–10 cm H_2_O, except in 4 spontaneously breathing patients receiving pressure support ventilation, as indicated in Additional file 1. These included 3 non-ARDS patients bridged for lung transplantation, and one ARDS patients who was placed under pressure support ventilation after 22 h of ECMO support. We did not specifically monitor patient effort in these 4 spontaneously breathing patients, e,g. by determining esophageal pressure (Pes), or by measuring P 0.1 (airway pressure during the first 100 msec of inspiration) [24]. During ECMO support, prone positioning of the patient was possible and indicated by the physicians in charge.

### Data collection

Demographic variables, Simplified Acute Physiology Score (SAPS 2), Sequential Organ Failure Assessment (SOFA) in the first 24 h, indications for ECMO, duration of mechanical ventilation before ECMO initiation, ECMO duration and weaning, use of rescue therapies (inhaled nitric oxide -iNO- and prone positioning -PP-), as well as length of ICU stay were collected. We also retrospectively calculated for each patient the Respiratory ECMO Survival Prediction (RESP) score [25]. We then determined for each patient the predicted survival (in percent) according to the RESP score. Arterial blood gas and lactate data were recorded just before (1-3 h), and 3 h after ECMO initiation. Ventilator settings and respiratory monitoring variables were collected just before, as well as 24 and 48 h after ECMO implantation, and included the mode of ventilation, tidal volume (Vt) normalized to predicted body weight (mL.kg^− 1^ PBW), RR (min^− 1^), peak airway pressure (PAwP), quasi static plateau pressure (dynamic Pplat, as inspiratory manual occlusions were not recorded) and PEEP, all pressures in cm H_2_O. From these data, we calculated the dynamic driving pressure (DP = dynamic Pplat-PEEP, cm H_2_O), quasi static respiratory system compliance (Vt/DP, mL.cm H_2_O^− 1^) and total Mechanical Power (MP), an index of the energy delivered to the respiratory system during MV. For patients under volume-controlled ventilation, MP was calculated according to Gattinoni’s simplified equation. [26, 27]: MP = 0.098 x RR x Vt x [PAwP − (0.5 x DP)]. For patients under pressure-controlled ventilation, we used the simplified equation developed by Becher et al. [28] and further validated by Chiumello et al. [29]: MP = 0.098 x RR x Vt x [PEEP + ΔPinsp], where ΔPinsp is the pressure (cmH_2_O) above PEEP during pressure-controlled ventilation. MP was expressed in J.min^− 1^.

### Presentation of data and statistical analysis

Continuous variables are expressed as medians and interquartile ranges (IQR) and categorical data as absolute numbers and percentages. Predicted survival according to the RESP score is presented as means±SD. The outcome of interest was ICU mortality. Data were compared between ICU survivors and non-survivors, both in the whole population and in the subgroup of ARDS patients, using the Wilcoxon’s rank sum test for continuous variables, and the chi-square test for categorical variables. We also compared data between ARDS and non-ARDS patients, regardless of the survival status, using the same statistical tests. To determine possible factors associated with mortality, we performed univariate logistic regression analyses (continuous variables), and contingency analyses with Pearson’s test (categorical variables). Variables associated with mortality in univariate analysis at a *p* value < 0.1 were then incorporated in a multivariate logistic regression model. Regarding respiratory variables, we included only DP in the multivariate analysis and omitted Pplat, PEEP and MP, which are mathematically coupled with DP. For all tests, a *p* value < 0.05 was considered significant. All analyses were performed using the JMP software, version 15 (SAS© Institute Inc., Cary, North Carolina, USA).

## Results

**Fig. 1:**
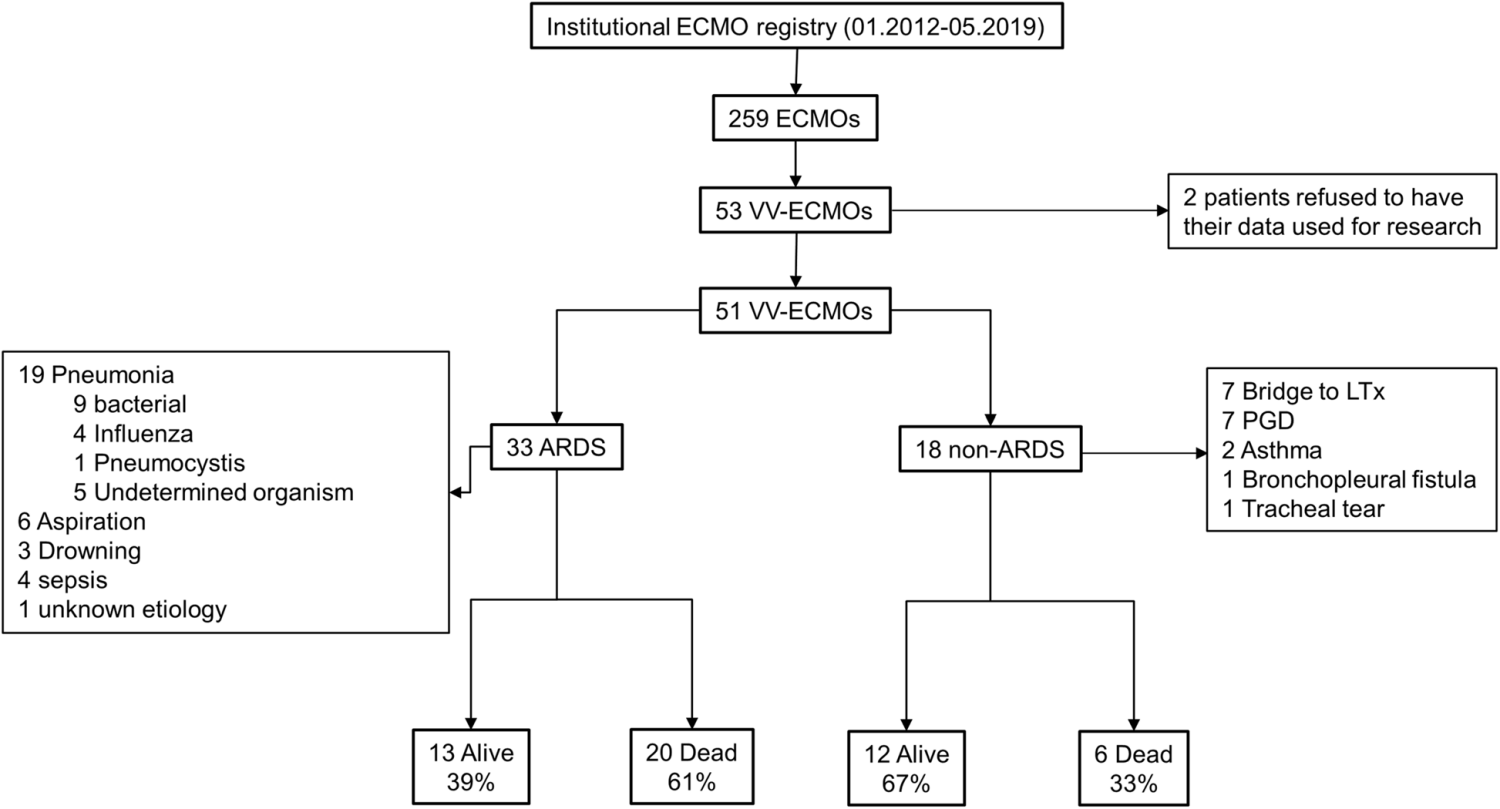
Study flowchart

From January 1st, 2012 to May 31st, 2019, 53 patients were treated with VV-ECMO, including 2 patients who specified their refusal to have their data used for research. Thus, the final cohort included 51 patients (see Additional file 2 for patients’ details), comprising 33 ARDS and 18 non-ARDS patients, as presented in the flowchart of Fig. 1. The characteristics of patients at baseline (before ECMO initiation) are shown in Table 1. In the whole cohort and in the ARDS cohort, survivors had a shorter duration of MV before ECMO and a higher RESP score with greater predicted survival. Table 2 presents the ECMO characteristics and ICU LOS in the whole population and in the ARDS cohort. The only significant difference was a more frequent weaning of ECMO in survivors. Comparison of baseline and ECMO characteristics between ARDS and non-ARDS patients, shown in Additional file 3, indicated that non-ARDS patients had lower SAPS 2 and SOFA scores, received less often rescue therapies before ECMO initiation (iNO and PP), and had a greater ICU survival, although their RESP score and predicted survival did not differ from patients in the ARDS cohort.

**Table 1:** Patient characteristics at baseline (before ECMO initiation)

Variable	Total cohort (*n=*51)	Dead (*n=*26)	Alive (*n=*25)	*p* value
VV-ECMO indication, n (%)				
ARDS	33 (65)	20 (61)	13 (39)	
Non-ARDS	18 (35)	6 (33)	12 (67)	
Age, y	56 (37-64)	58 (39-65)	48 (35-60)	0.346
Female Gender, n (%)	18 (35)	19 (37)	14 (27)	0.202
SAPS 2	47 (36-64)	55 (39-65)	43 (32-62)	0.247
SOFA, first 24h	11.0 (8.0-13.0)	10.5 (8.0-15.0)	11.0 (8.0-12.0)	0.317
MV before ECMO, days	2.0 (0.3-5.5)	3.2 (0.5-8.1)	0.5 (0.3-2.6)	0.014*
P/FO_2_	65 (52-95)	61 (51-84)	83 (54-118)	0.156
iNO, n (%)	24 (47)	12 (46)	12 (48)	0.895
PP, n (%)	13 (25)	9 (35)	4 (16)	0.127
RESP Score	0.0 (-3.0-3.0)	-2.5 (-4.0-0.8)	2.0 (-0.5-4.0)	0.001*
Predicted survival, %	52±22	43±19	62±22	0.004*
ICU survival, n (%)	25/51 (49)			
	ARDS cohort (*n=*33)	ARDS dead (*n=*20)	ARDS alive (*n=*13)	*p* value
Age, y	59 (32-67)	59.5 (31.3-69.0)	45.0 (32.0-66.5)	0.579
Female Gender, n (%)	10 (30)	6 (30)	9 (31)	0.963
SAPS 2	56 (43-69)	56 (45-66)	56 (42-74)	0.971
SOFA, first 24h	11.0 (9.0-13.0)	11.0 (8.5-14.5)	11.0 (10.0-12.5)	0.824
MV before ECMO, days	2.5 (0.4-7.6)	4.9 (0.7-9.3)	0.5 (0.3-2.6)	0.013*
P/FO_2_	62 (52-87)	62 (51-87)	65 (52-87)	0.941
iNO, n (%)	19 (58)	10 (50)	9 (69)	0.275
PP, n (%)	12 (36)	9 (45)	3 (23)	0.201
RESP Score	-1.0 (-3.0-3.0)	-3.0 (-4.25-0.25)	3.0 (0.0-4.0)	0.006*
Predicted survival, %	51±24	43±20	64±24	0.016*
ICU survival, n (%)	20/33 (39)			

Continuous data expressed as medians (interquartile range), except predicted survival (means±SD). The * symbol indicates significant differences (*p* < 0.05)

*Abbreviations*. *ARDS *Acute respiratory distress syndrome, *ICU *Intensive Care Unit, *iNO *inhaled nitric oxide, *MV *Mechanical ventilation, *P/FO*_2 _ Arterial partial pressure of oxygen/inspired oxygen fraction, *PP *Prone positioning, *RESP Score *Respiratory extracorporeal membrane oxygenation survival prediction score, *SAPS 2 *Simplified acute physiological score 2, *VV-ECMO *Veno-venous extracorporeal membrane oxygenation, *y *years

**Table 2:** ECMO characteristics and length of ICU stay

Variable	Total cohort (*n=*51)	Dead (*n=*26)	Alive (*n=*25)	*p* value
ECMO duration, days	8.2 (4.2-15.2)	11.4 (2.8-19.9)	6.6 (4.2-10.5)	0.258
ECMO weaning, n (%)	34 (65)	9 (35)	25 (100)	<0.001*
iNO on ECMO, n (%)	20 (39)	10 (38)	10 (40)	0.910
PP on ECMO, n (%)	4 (8)	2 (8)	2 (8)	0.967
LOS ICU, days	22.4 (13.8-37.2)	23.4 (10.5-37.1)	21.0 (15.8-49.0)	0.270
Variable	ARDS cohort (*n=*33)	ARDS dead (*n=*20)	ARDS alive (*n=*13)	*p* value
ECMO duration, days	8.3 (3.6-16.1)	11.4 (2.3-18.7)	6.7 (4.0-13.5)	0.685
ECMO weaning, n (%)	20 (61)	7 (35)	13 (100)	<0.001*
iNO per ECMO, n (%)	13 (39)	8 (40)	5 (39)	0.929
PP per-ECMO, n (%)	4 (12)	2 (10)	2 (15)	0.643
LOS ICU, days	18.7 (11.5-36.7)	20.6 (9.0-36.2)	18.5 (13.7-50.4)	0.507

Continuous data expressed as median (interquartile range). The * symbol indicates significant differences (*p* < 0.05)

*Abbreviations*. *ARDS *Acute respiratory distress syndrome, *ECMO *Extracorporeal membrane oxygenation, *ICU *Intensive Care Unit, *iNO *inhaled nitric oxide, *LOS *Length of stay, *PP *Prone positioning

The results of arterial blood gas analyses (pre-ECMO and at 3 h on ECMO) are shown in Additional file 4. No significant differences were noted between survivors and non-survivors in any of the recorded variables at the two time-points. Comparisons between ARDS and non-ARDS patients only showed that non-ARDS patients displayed higher PaO_2_ and SaO_2_ at 3 h on ECMO (Additional file 5). Table 3 presents the results of ventilatory settings and monitoring variables at 3 time-points, just before ECMO (pre-ECMO) and at 24 and 48 h on ECMO. When considering all patients, pre-ECMO results were comparable between survivors and non-survivors. In contrast, survivors had lower Pplat and DP, as well as higher C_RS_ at 24 and 48 h on ECMO. In ARDS patients, survivors had a higher C_RS_ and PEEP before ECMO. On ECMO, survivors had lower DP (24 h), as well as higher C_RS_ (at 24 and 48 h). Comparison between ARDS and non-ARDS patients (Additional file 6) showed that non-ARDS patients had lower Vt, PEEP and MP before ECMO, and a lower PEEP and MP on ECMO (at 24 and 48 h). The ventilatory strategy before and on ECMO did not significantly vary over time during the period of the study, both in the whole and in the ARDS populations, as indicated in Additional files 7 and 8.

**Table 3:** Ventilatory settings and monitoring variables

Variable	All cohort (*n=*51)	Dead (*n=*26)	Alive (*n=*25)	*p* value
*Pre-ECMO*				
Vt (ml/kg PBW)	5.6 (4.8-6.6)	5.3 (4.1-6.2)	5.8 (4.9-6.6)	0.209
Pplat (cm H_2_O)	29.0 (25.3-34.0)	29.0 (25.0-34.0)	29.0 (24.5-34.5)	0.916
DP (cm H_2_O)	19.5 (16.0-27.0)	21.0 (17.0-27.0)	17.0 (14.0-26.5)	0.216
C_RS_ (ml/cm H_2_O)	19.3 (12.0-27.3)	14.9 (10.8-22.8)	21.9 (13.6-29.4)	0.113
RR (min^-1^)	24.0 (20.0-28.0)	25.0 (20.0-29.5)	24.0 (19.0-26.3)	0.279
PEEP (cm H_2_O)	8.0 (5.0-10.0)	7.0 (5.0-9.0)	8.0 (4.0-13.5)	0.376
Power (J/min)	23.8 (16.2-30.3)	21.8 (15.9-26.2)	25.7 (16.5-34.7)	0.192
*24h on ECMO*				
Vt (ml/kg PBW)	3.5 (2.5-4.3)	3.1 (2.3-4.1)	3.8 (2.9-4.4)	0.347
Pplat (cm H_2_O)	23.0 (20.0-26.0)	25.5 (21.0-29.5)	21.5 (19.0-23.8)	0.019*
DP (cm H_2_O)	15.0 (11.3-19.0)	17.0 (13.0-20.8)	12.5 (10.0-17.8)	0.024*
C_RS_ (ml/cm H_2_O)	15.5 (10.2-21.9)	12.1 (8.5-17.2)	19.3 (13.9-26.7)	0.010*
RR (min^-1^)	14.5 (10.0-15.0)	15.0 (11.5-16.0)	13.0 (10.0-15.0)	0.233
PEEP (cm H_2_O)	7.0 (5.0-10.0)	7.5 (5.0-10.0)	7.0 (5.0-12.0)	0.680
Power (J/min)	5.6 (3.5-7.5)	5.5 (3.3-7.4)	5.6 (3.8-7.8)	0.815
*48h on ECMO*				
Vt (ml/kg PBW)	4.2 (2.8-5.3)	3.8 (2.1-4.7)	4.6 (3.4-5.5)	0.074
Pplat (cm H_2_O)	23.0 (19.3-26.8)	24.0 (21.0-29.5)	21.0 (19.0-25.0)	0.018*
DP (cm H_2_O)	14.0 (12.0-17.5)	14.0 (13.0-23.0)	12.0 (10.0-16.0)	0.014*
C_RS_ (ml/cm H_2_O)	17.3 (11.3-26.9)	11.8 (7.5-21.0)	22.6 (16.6-31.7)	0.003*
RR (min^-1^)	15.0 (11.0-16.0)	15.0 (14.0-15.3)	13.0 (10.0-16.0)	0.277
PEEP (cm H_2_O)	8.0 (5.0-10.0)	8.0 (5.0-10.0)	8.0 (5.0-11.0)	0.609
Power (J/min)	6.3 (5.1-10.4)	6.1 (3.9-11.4)	6.7 (5.2-8.9)	0.613
	ARDS cohort (*n=*33)	ARDS dead (*n=*20)	ARDS alive (*n=*13)	*p* value
*Pre-ECMO*				
Vt (ml/kg)	5.8 (5.0-7.1)	5.8 (4.8-7.1)	6.4 (5.5-7.6)	0.239
Pplat (cm H_2_O)	29.0 (26.0-34.0)	29.0 (25.0-33.0)	30.0 (26.3-35.8)	0.489
DP (cm H_2_O)	18.0 (16.0-25.0)	20.0 (17.0-27.0)	17.0 (14.5-20.3)	0.073
C_RS_ (ml/cm H_2_O)	21.4 (14.2-28.9)	16.1 (10.9-22.8)	24.1 (21.2-31.3)	0.043*
RR (min^-1^)	24.0 (20.0-28.0)	25.0 (20.0-30.0)	24.0 (18.0-24.5)	0.072
PEEP (cm H_2_O)	8.0 (5.0-14.0)	7.0 (5.0-9.0)	11.0 (8.3-17.5)	0.007*
Power (J/min)	25.6 (17.4-30.9)	24.4 (17.4-29.6)	27.1 (19.1-37.2)	0.168
*24h on ECMO*				
Vt (ml/kg)	3.6 (2.5-4.5)	3.6 (2.5-4.5)	3.7 (2.9-4.9)	0.774
Pplat (cm H_2_O)	23.0 (20.3-27.8)	26.0 (21.0-30.0)	22.0 (19.0-24.5)	0.148
DP (cm H_2_O)	14.0 (11.0-18.0)	16.0 (13.0-20.0)	11.0 (8.0-15.0)	0.020*
C_RS_ (ml/cm H_2_O)	18.0 (10.5-25.2)	12.6 (9.7-19.7)	22.3 (18.8-28.9)	0.025*
RR (min^-1^)	14.0 (10.5-15.0)	15.0 (13.0-16.0)	13.0 (10.0-15.0)	0.226
PEEP (cm H_2_O)	9.5 (6.3-12.0)	8.0 (6.0-10.0)	12.0 (6.5-13.0)	0.132
Power (J/min)	6.1 (4.2-8.4)	6.2 (4.7-9.4)	5.9 (3.9-8.3)	0.803
*48h on ECMO*				
Vt (ml/kg)	4.3 (3.2-5.5)	4.1 (2.2-5.4)	4.7 (3.8-5.5)	0.313
Pplat (cm H_2_O)	23.5 (21.0-26.8)	24.0 (21.0-28.0)	23.0 (20.3-26.0)	0.261
DP (cm H_2_O)	14.0 (12.0-16.0)	14.0 (13.0-18.8)	12.5 (9.0-16.0)	0.107
C_RS_ (ml/cm H_2_O)	19.1 (11.9-28.5)	16.1 (8.5-26.1)	24.8 (18.3-31.0)	0.037*
RR (min^-1^)	15.0 (12.0-16.0)	15.0 (14.0-15.8)	13.0 (10.5-16.0)	0.286
PEEP (cm H_2_O)	8.0 (7.0-12.0)	8.0 (7.0-10.8)	10.0 (7.5-13.0)	0.268
Power (J/min)	7.7 (5.3-11.6)	6.8 (5.1-12.1)	8.6 (5.4-10.6)	0.816

All data expressed as median (interquartile range). ﻿The * symbol indicates significant differences (*p* < 0.05)

*Abbreviations*. *ARDS *Acute Respiratory Distress Syndrome, *Crs *Respiratory System Compliance, *DP *Driving Pressure, *PBW *Predicted Body Weight, *PEEP *Positive End-Expiratory Pressure, *Pplat *Plateau Pressure, *RR *Respiratory Rate, *Vt *tidal volume

Results of univariate analyses are shown in Additional file 9, and those of multivariate analyses are shown in Table 4. In the whole population, variables associated with mortality in univariate analysis included Pplat, DP and C_RS_ on ECMO (24 and 48 h). In ARDS patients, the duration of ventilation before ECMO, pre-ECMO PEEP, as well as DP and C_RS_ at 24 h on ECMO were associated with mortality. Multivariate regression in the total cohort revealed that ECMO indication (ARDS vs. non-ARDS) and a higher DP at 24 h on ECMO were independently associated with mortality. In the ARDS cohort, variables associated with mortality included a longer duration of MV before ECMO and a higher DP at 24 h on ECMO.

**Table 4:** Variables associated with ICU mortality: multivariate analysis

All patients		
Variable	OR	*p* value
non-ARDS vs ARDS	0.03 [0.00-0.48]	0.002*
P/FO_2_ pre-ECMO	0.98 [0.95-1.01]	0.108
DP 24h on ECMO	1.41 [1.06-1.86]	0.005*
DP 48h on ECMO	1.10 [0.94-1.29]	0.195
ARDS patients		
Variable	OR	*p* value
Days MV pre-ECMO	1.56 [1.03-3.03]	0.033*
DP pre-ECMO	1.02 [0.75-1.42]	0.891
DP 24h on ECMO	1.47 [1.02-2.74]	0.034*
DP 48h on ECMO	1.03 [0.57-1.83]	0.928

*Abbreviations*: *ARDS *Acute Respiratory Distress Syndrome, *DP *Driving pressure, *ECMO *Extracorporeal membrane oxygenation, *MV *Mechanical ventilation, *OR *Odds ratio, *P/FO*_2 _Arterial partial pressure of oxygen/inspired oxygen fraction. ﻿The * symbol indicates significant differences (*p* < 0.05)

**Fig. 2:**
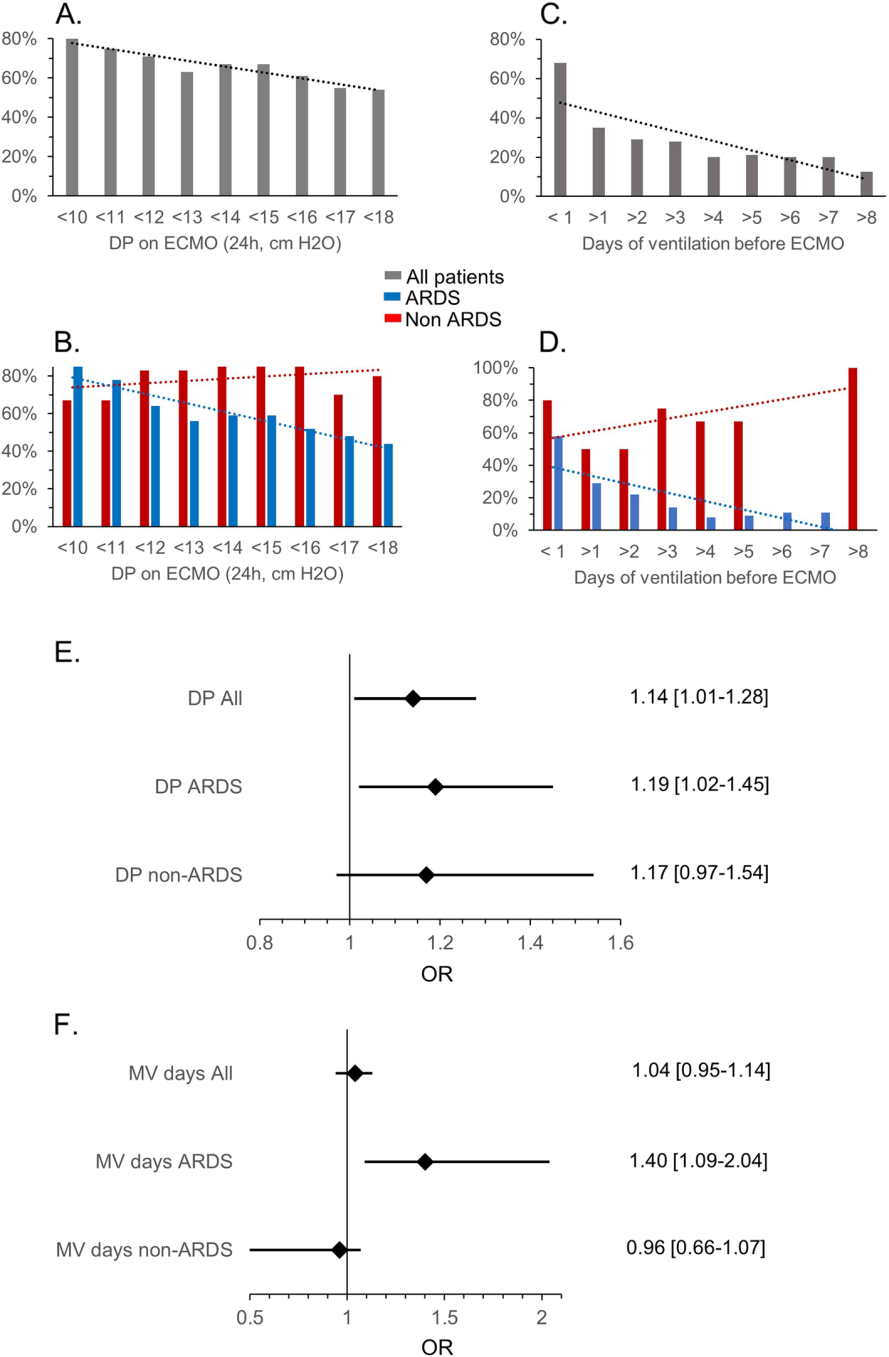
Prognostic role of Driving Pressure on ECMO and of pre-ECMO MV duration in VV-ECMO for ARDS and non-ARDS indications. **A**, **B**. Survival according to DP at 24 h ECMO in the whole cohort (**A**) and in the ARDS and non-ARDS cohorts (**B**). **C**, **D**. Survival according to MV duration before ECMO in the whole cohort (**C**) and in the ARDS and non-ARDS cohorts (**D**). **E**, **F**. Odds ratios for mortality (univariate analysis) predicted by DP at 24 h ECMO (**E**) and by days of MV before ECMO (**F**) in the whole cohort, ARDS and non-ARDS cohorts

To highlight the prognostic implication of MV duration before ECMO and of DP on ECMO, we determined the survival rate in the ARDS, non-ARDS and whole cohort of patients according to the values of DP and days of MV before ECMO implantation in each population. As illustrated in Fig. 2, there was a progressive decline in survival with increasing DP at 24 h on ECMO (Fig. 2A), an effect that was noted only in ARDS, but not in non-ARDS population (Fig. 2B). We also noted a steady decline in survival according to the number of MV days before ECMO in the whole population (Fig. 2C), which was related to a major effect in ARDS patients, whereas it was not the case in non-ARDS patients (Fig. 2D). The impact of 1 unit variation of each variable on mortality is represented in the forest plot graphs shown in Fig. 2E, F, depicting the odds ratios and 95% CI for mortality in univariate regression analysis. In the whole cohort and in ARDS patients, the probability of death significantly increased, respectively by 14% and 19%, for each 1 cm H_2_O increase of DP. No significant influence of DP on mortality was noted in non-ARDS patients. Regarding MV duration, a significant association was only found in ARDS patients, in whom each additional day of MV before ECMO increased the probability of death by 40%.

## Discussion

The main results of our study indicate that, in an unselected population of patients treated with VV-ECMO, the main predictive factors for ICU mortality were the indication for ECMO, and, in ARDS patients, the duration of MV before ECMO initiation, as well as the value of driving pressure after 24 h on ECMO support.

We included all consecutive patients treated with VV-ECMO in the pre-COVID era over a 7-year period, regardless of the etiology of acute respiratory failure (ARDS and non-ARDS), age and the timing of ECMO initiation. Although the overall ICU survival of our whole cohort (49%) was close to the predicted survival according to the RESP score (52%), it was lower than predicted in the ARDS subgroup (39% vs. 51% predicted). In addition, survival in our ARDS patients was lower than that reported in the two landmark clinical trials on ECMO in ARDS, EOLIA (65% survival) [14] and CESAR (63% survival) [13], which could be partly explained by the exclusion of patients older than 65 years and of patients ventilated for more than 7 days in both trials, whereas we included such patients. In addition, prone positioning, known to improve survival in severe ARDS [30], had been applied in only 36% of ARDS patients of our cohort before ECMO, contrasting with 56% in the EOLIA trial. By contrast, our results compare well with those of several observational studies on VV-ECMO in non-COVID ARDS, reporting hospital survival between 30 and 46% [4, 5, 31, 32]. Therefore, although survival in our cohort was lower than that of major RCTs, it may be considered as representative of the outcome of VV-ECMO in a real-world clinical context.

In non-ARDS patients, primary indications were bridge to transplantation (BTT, *n* = 7) and bridge to recovery (BTR, *n* = 7) for severe PGD after LTx. In BTT indications, 4/7 patients (57%) were eventually transplanted, who all (100%) survived the ICU stay, which agrees with the reports by Tipograf et al. (59% successful bridge, 88% survival) [10] and Biscotti et al. (56% bridge, 92% survival) [9]. Therefore, if patients can be successfully bridged to LTx, preoperative VV-ECMO appears associated with excellent survival, as recently emphasized in cohort studies showing comparable post-LTx outcomes between bridged and non-bridged patients [33, 34]. Regarding BTR indications for severe PGD, only few data are available regarding its impact on outcome. In our cohort, death occurred in 3/7 patients (43%), which is slightly higher than in two recent registries reporting hospital and 90-day mortality of 25% and 33%. This difference could be partly explained by the severe post-transplant hemorrhagic complications responsible of the death of 2 of our patients. Other indications for VV-ECMO in our study included asthma (2 patients) and refractory air leaks (2 patients), who all survived to ICU discharge, consistent with previous reports showing VV-ECMO survival rates of about 90% in asthma [7, 8] and up to 100% in bronchopleural fistula [35].

When assessing factors associated with mortality, the indication for ECMO (ARDS vs. non-ARDS) was found to be independently linked to mortality in multivariate analysis, which agrees with previous data on VV-ECMO survival in mixed populations of patients with acute respiratory failure [36]. Non-ARDS patients had an ICU survival of 67%, contrasting with 39% in ARDS patients, which can be explained by three elements. First, non-ARDS patients were less critically ill, as indicated by lower SAPS 2 and SOFA scores during the first 24 h of admission. In contrast, the RESP scores of the two groups were comparable, with similar predicted survival (51%, ARDS, 54%, non-ARDS). It is here worth to mention that most non-ARDS patients in our cohort underwent VV-ECMO before or after LTx, and such patients were not specifically included in the model applied to develop the RESP score [25, 37]. In addition, in a recent study evaluating several mortality prediction scores, the RESP score had only a moderate discriminative performance [38]. Second, patients in the non-ARDS group suffered from pathologies more rapidly reversible than ARDS (e.g. asthma) or amenable to definitive therapy (transplantation). Third, non-ARDS patients had better improvements of oxygenation indices (PaO_2_, SaO_2_) upon ECMO implantation, and also displayed lower total mechanical power than ARDS patients under ECMO, hence, may have been at lesser risk of ongoing lung injury after ECMO initiation.

A second major factor associated with mortality was the delay to ECMO implantation in ARDS patients. Indeed, the probability of death increased by 40% for each additional day of MV before ECMO. The major impact of MV duration before ECMO in ARDS has been highlighted by previous authors. In the pre-COVID era, Brogan et al. reported that the median MV duration before ECMO was 42 h in survivors and 65 h in non-survivors (12 vs. 76 h in our own study), and this factor was independently associated with mortality in multivariate analysis [36]. Wu et al. found a linear increase of mortality with each additional day of MV before ECMO in ARDS and suggested that a 7-day delay should be the upper limit beyond which ECMO should probably not be implanted [39]. Comparable findings have been more recently reported in studies on VV-ECMO for COVID-19 ARDS [40, 41], although some authors did not find such an association [42]. An analysis of 4812 patients from the ELSO registry indicated that each doubling of the number of hours of MV before ECMO correlated to about a 5% increase of the hazard ratio for mortality [43]. The association between longer MV duration before ECMO and mortality in COVID-19 has been also highlighted in a recent meta-analysis of 42 observational studies [19]. Ongoing VILI, the consequences of protracted hypoxemia and high FiO_2_, pulmonary circulatory dysfunction, as well as prolonged use of sedative and paralyzing agents, may all be factors underlying the negative impact of delaying ECMO initiation in ARDS. Therefore, the current criteria for ECMO implantation, primarily based on the severity of hypoxemia, may be too stringent and might be reassessed, as underscored in a recent editorial on this issue [44].

The ventilation strategy to apply during VV-ECMO is presently debated [45]. Common practice is to reduce V_T_ at 4–6 ml/kg PBW and maintain a PEEP of at least 10 cm H_2_O, in order to keep Pplat and DP below 24 and 14 cm H_2_O, respectively, as reported in the EOLIA trial. A strategy of “ultraprotective” ventilation with further reductions of V_T_ and DP has been advocated, as it might reduce further the risk of VILI [22]. It has been notably shown in a pig model of ARDS and VV-ECMO that near apneic ventilation could reduce lung histological alterations [46]. In human ARDS, a study found that reducing DP during VV-ECMO using continuous positive airway pressure (CPAP) led to a decrease of plasma levels of several inflammatory biomarkers, even in patients with very low baseline V_T_ [47]. In contrast, a recent study by Guervilly et al. did not confirm such results when applying ultraprotective ventilation, but in this study, baseline DP was low (10 cm H_2_O) and was not significantly affected by the reduction of V_T_ [2]. Therefore, it is possible that ultraprotective ventilation might only benefit patients with the most severely reduced respiratory system compliance, who continue to display tidal hyperinflation despite low volume ventilation.

In our study, we found that DP under ECMO was significantly greater in non-survivors, an effect due to the major impact of elevated DP in ARDS, but not in non-ARDS patients. This concurs with Serpa Neto et al. who found in an analysis of 9 studies totalizing 545 patients with H1N1 ARDS treated with VV-ECMO, that DP during the first days of ECMO was the only ventilatory parameter independently associated with in-hospital mortality [20]. We determined that the probability of death raised by 19% for each cm H_2_O increase of DP at 24 h on ECMO. This finding is close to the results of a recent study by Magunia et al., who reported that DP at 12 h on ECMO in ARDS patients was predictive of mortality with an odds ratio of 1.25 [48]. In contrast, such an association of DP with mortality was not found in the 2020 LIFEGARDS cohort study evaluating mechanical ventilation strategies in a multicenter international cohort of 350 ARDS patients [21], which could reflect the adoption of more protective ventilatory strategies in recent years.

The higher DP in non-survivors was not related to greater Vt and Pplat, but to a significantly lower respiratory system compliance. It was not either related to differences in PEEP levels under ECMO, which differs from results by Schmidt et al., who reported higher PEEP under ECMO in survivors [49]. One may argue, therefore, that higher DP during ECMO was simply a proxy of more severe ARDS, hence, associated with lesser chances of survival. Non-survivors indeed exhibited lower compliance and were administered significantly lower PEEP levels before ECMO, pointing to more severe pulmonary involvement. This is consistent with the lower pre-ECMO PEEP and Crs in ARDS non-survivors reported in two large retrospective and prospective databases [21, 49]. By reflecting the severity and progression of the disease, a higher DP on ECMO could help identify different phenotypes of patients, who might therefore benefit from a personalized ultraprotective mode of ventilation, as discussed above.

At variance with DP, mechanical power (MP) did not significantly differ between survivors and non-survivors. MP represents a unifying variable measuring the energy delivered by ventilation to the respiratory system, which has been associated with mortality in ARDS. During ECMO, MP markedly decreases, primarily as a result from the reduction of Vt [45], which may then limit its prognostic significance in this setting. Only a few studies addressed this issue, with conflicting results. While Chiu et al. reported a significant association between MP in the early phase of ECMO and mortality [50], Belliato et al. [51] and Schmidt et al. [21], did not report such an association. Therefore, it is possible that under VV-ECMO, DP may be more discriminant than MP to track ongoing VILI. Additional studies are therefore needed to solve this question.

Our study has obvious limitations related, first, to its retrospective nature and limited sample size. Second, it reflects, at least in part, some earlier practice which evolved over the past years with the refinement in the clinical management of VV-ECMO patients, especially regarding mechanical ventilation settings. Third, we only evaluated ICU mortality and can therefore not conclude on longer term survival. Fourth, having analyzed a cohort treated before 2020, our findings may not apply to patients treated with VV-ECMO for COVID-19 ARDS. Fifth, we did not specifically monitor patient efforts in the 4 spontaneously breathing patients under ECMO (3 non-ARDS and 1 ARDS patient). Spontaneous breathing may cause or aggravate lung injury in ARDS through excessive increase in transpulmonary pressure and unsuspected overstretch, even after adequate CO_2_ removal under full ECMO support [21, 52]. It is therefore advocated to monitor patient effort during spontaneous breathing, notably by computing transpulmonary and inspiratory muscle pressures from the measurement of esophageal pressure, as well as respiratory drive from the measurement of P 0.1 [24].

Finally, our data confirm some earlier findings [19, 20, 43, 48], and may therefore be criticized for a lack of novelty. However, we believe that the current results still provide some interesting insights for the management of patients under VV-ECMO. Current recommendations indicate to target a Vt < 4 ml/kg and a DP < 14 cm H_2_O during VV-ECMO in ARDS patients [3, 21, 45]. The major prognostic impact of driving pressure after 24 h of ECMO reported in our study suggests that a strategy of ultraprotective ventilation, or even near apneic ventilation [45], could be applied already at the very early stage of ECMO to the subset of patients with the most advanced form of lung injury. Also, the significant association of the delay to ECMO implantation with mortality in our study, suggests that the criteria for ECMO initiation could incorporate some aspects of respiratory mechanics (most notably compliance) for an earlier implementation in some patients.

In conclusion, in non-selected patients with acute respiratory failure treated with VV-ECMO before the COVID pandemic in a medium-size volume center, ICU survival was 39% in ARDS and 67% in non-ARDS patients. Besides the indication of VV-ECMO, key prognostic factors in ARDS patients were the value of driving pressure in the early phase of ECMO support (24 h), and the delay to ECMO initiation. These data suggest, first, that ultraprotective ventilation might benefit patients with persisting lung stress in spite of low tidal volume ventilation during VV-ECMO and, second, that the criteria for VV-ECMO initiation in ARDS might be modified for an earlier detection of patients susceptible to benefit from the extracorporeal support. These two hypotheses should be addressed in future studies on VV-ECMO in ARDS.

### Supplementary Information

**Additional file 1.** Type of ventilatory support.

**Additional file 2.** Patient details.

**Additional file 3.** Baseline and ECMO characteristics in ARDS and non-ARDS patients.

**Additional file 4.** Arterial blood gas data.

**Additional file 5.** Arterial blood gas data in ARDS and non-ARDS patients.

**Additional file 6.** Ventilator settings and monitoring variables in ARDS and non-ARDS patients.

**Additional file 7.** Ventilatory strategies during the 8 years of the study in the whole population. Patients were grouped in 4 periods of 2 y each. Differences across years were analyzed by ANOVA.

**Additional file 8.** Ventilatory strategies during the 8 years of the study in the ARDS population. Patients were grouped in 4 periods of 2 y each. Differences across years were analyzed by ANOVA.

**Additional file 9.** Variables associated with ICU mortality: univariate analysis.

## Data Availability

All data generated or analysed during this study are included in this article and its supplementary information files.

## Abbreviations

ARDS: Acute Respiratory Distress Syndrome
Crs: Respiratory System Compliance
DP: Driving Pressure
ELSO: Extracorporeal Life Support Organization
FiO_2_: Inspired Fraction of Oxygen
ICU: Intensive Care Unit
IQR: Interquartile Range
iNO: Inhaled Nitric Oxide
LOS: Length Of Stay
LTx: Lung Transplantation
MV: Mechanical Ventilation
MP: Mechanical Power
PAwP: Peak Airway Pressure
PBW: Predicted Body Weight
PEEP: Positive End-Expiratory Pressure
PGD: Primary Graft Dysfunction
Pplat: Plateau Pressure
PP: Prone Positioning
RESP Score: Respiratory ECMO Survival Prediction Score
RR: Respiratory Rate
SAPS: Simplified Acute Physiology Score
SOFA: Sequential Organ Failure Assessment
VILI: Ventilator-Induced Lung Injury
VV-ECMO: Veno-Venous Extra-Corporeal Membrane Oxygenation
