# Supplementary material for: Retrospective analysis of factors associated with outcome in veno-venous extra-corporeal membrane oxygenation
Source: BMC Pulm Med. 2023 Aug 16;23:301. doi: 10.1186/s12890-023-02591-5 (PMC10429070; doi:10.1186/s12890-023-02591-5)
Supplement: Supplementary file 1 — Additional file 1. Type of ventilatory support. [file 12890_2023_2591_MOESM1_ESM.docx]

Additional File 1. Type of ventilatory support

Before ECMO (n=51) 24h on ECMO (n=50) 48h on ECMO (n=48)

ARDS non ARDS ARDS non ARDS ARDS non ARDS Ventilatory support (n = 33) (n = 18) (n = 32) (n = 18) (n = 30) (n = 18)

Volume-controlled 30 12 28 13 25 15

Pressure-controlled 3 3 3 2 3 1

Pressure-support 0 0 1 1 1 0

NIV 0 2 0 2 0 2

HFNO 0 1 0 0 0 0

Oxygen Mask 0 0 0 0 1 0

NIV: Non-Invasive Ventilation; HFNO: High Flow Nasal Oxygen
