# Supplementary material for: Retrospective analysis of factors associated with outcome in veno-venous extra-corporeal membrane oxygenation
Source: BMC Pulm Med. 2023 Aug 16;23:301. doi: 10.1186/s12890-023-02591-5 (PMC10429070; doi:10.1186/s12890-023-02591-5)
Supplement: Supplementary file 2 — Additional file 2. Patient details. [file 12890_2023_2591_MOESM2_ESM.docx]

Additional File 2. Patient details

Sex, age Indication for VV-ECMO pre-ECMO (d) on ECMO (d) Weaning Outcome

M, 57 ARDS - Pneumonia, bacterial 2.5 16 Y Dead

M, 19 ARDS - Pneumonia, undetermined 22.9 13.3 N Dead

M, 64 PGD, unilateral LTx 5.5 13.1 Y Dead

F, 36 Bridge to LTx, transplanted 44.8 43.3 Y Alive

F, 60 PGD, bilateral LTx 0.1 6 Y Alive

M, 53 ARDS - Pneumonia, influenza 9.6 8.2 N Dead

M, 37 PGD, unilateral LTx 0.3 0.4 N Dead

M, 20 ARDS - Pneumonia, bacterial 8 48.4 N Dead

F, 46 PGD, bilateral LTx 2 7.2 Y Alive

M, 21 ARDS - Sepsis 0.3 3.3 Y Alive

F, 34 Bridge to LTx (CLAD), transplanted 3.8 14.9 Y Alive

F, 24 ARDS - Pneumonia, bacterial 11.2 8.3 N Dead

M, 43 PGD, bilateral LTx 0.2 5.7 Y Alive

F, 56 ARDS - Pneumonia, bacterial 0.3 2 N Dead

F, 65 ARDS - Sepsis 3.1 7 Y Alive

M, 47 Bridge to LTx (IPF), not transplanted 3 34.1 N Dead

M, 66 ARDS - Pneumonia, undetermined 8.2 16.1 N Dead

M, 72 ARDS - Pneumonia, undetermined 3.4 23.3 N Dead

M, 59 Bridge to LTx (IPF), transplanted 0.5 3.4 Y Alive

M, 62 Bridge to LTx (IPF), not transplanted 2.2 23.9 N Dead

M, 61 ARDS - Aspiration 5.6 0.8 N Dead

M, 70 ARDS - Sepsis 7.9 19.5 Y Dead

F, 38 ARDS - Pneumonia, influenza 0.3 9.2 Y Alive

F, 70 ARDS - Pneumonia, influenza 2.6 11 N Dead

M, 72 ARDS - Aspiration 0.2 3.8 Y Alive

F, 60 ARDS - Aspiration 0.5 3 Y Dead

M, 60 ARDS - Aspiration 2.5 4.2 Y Alive

F, 54 PGD, bilateral LTx 0.1 11 Y Dead

M, 19 ARDS - Drowning 0.1 0.7 N Dead

M, 72 ARDS - Sepsis 4.1 12.5 Y Dead

F, 59 ARDS - Aspiration 0.4 1.3 N Dead

M, 68 ARDS - Pneumonia, influenza 2 37.6 Y Alive

M, 62 ARDS - Pneumonia, *P.jiroveci* 11.3 21.1 N Dead

M, 40 Bridge to LTx (IPF), not transplanted 1.1 8.8 N Dead

M, 31 Asthma 0.5 4.4 Y Alive

F, 16 ARDS - Unknown etiology 2.6 42 Y Alive

M, 72 ARDS - Pneumonia, undetermined 6 11.7 Y Dead

F, 59 PGD, bi-LTx 0.1 6.6 Y Alive

M, 45 ARDS - Pneumonia, bacterial 7.2 11.8 Y Alive

M, 64 ARDS - Pneumonia, undetermined 11.2 24.8 Y Dead

M, 72 ARDS - Pneumonia, bacterial 1.1 6.7 Y Alive

F, 68 Tracheal tear 0.3 2.8 Y Alive

F, 59 ARDS - Aspiration 0.2 4.2 Y Alive

M, 41 ARDS - Pneumonia, bacterial 0.5 5.9 Y Alive

F, 32 ARDS - Drowning 0.4 2.5 Y Alive

M, 18 ARDS - Drowning 0.1 1.9 Y Dead

M, 55 Bridge to LTx (IPF), transplanted 0.9 6.2 Y Alive

M, 48 Bronchopleural fistula 5.4 7.8 Y Alive

M, 54 ARDS - Pneumonia, bacterial 1.2 6.9 N Dead

F, 57 Asthma 0.3 8.9 Y Alive

M, 32 ARDS - Pneumonia, bacterial 0.1 15.2 Y Alive

CLAD: Chronic Lung Allograft Dysfunction; IPF: Interstitial Pulmonary Fibrosis; LTx: Lung Transplantation; PGD: Primary Graft Dysfunction
