# Supplementary material for: Retrospective analysis of factors associated with outcome in veno-venous extra-corporeal membrane oxygenation
Source: BMC Pulm Med. 2023 Aug 16;23:301. doi: 10.1186/s12890-023-02591-5 (PMC10429070; doi:10.1186/s12890-023-02591-5)
Supplement: Supplementary file 3 — Additional file 3. Baseline and ECMO characteristics in ARDS and non-ARDS patients. [file 12890_2023_2591_MOESM3_ESM.docx]

Additional File 3. Baseline and ECMO characteristics in ARDS and non-ARDS patients

Variable ARDS (n=33) Non-ARDS (n=18) p value

Baseline

Age, y 59 (32-67) 51 (39-59) 0.424

Female Gender, n (%) 10 (33) 8 (44) 0.313

SAPS 2 56 (43-69) 36 (30-45) 0.001*

SOFA, first 24h 11.0 (9.0-13.0) 8.5 (5.8-12.3) 0.045*

P/FO_2_ 62 (52-87) 93 (51-171) 0.086

MV before ECMO, days 2.5 (0.4-7.6) 0.7 (0.3-3.2) 0.133

iNO before ECMO, n (%) 19 (58) 5 (28) 0.042*

PP before ECMO, n (%) 12 (36) 1 (6) 0.016*

RESP Score -1.0 (-3.0-3.0) 1.0 (-1.25-2.0) 0.384

Predicted survival, % 51±24 54±20 0.631

ICU survival, n (%) 13/33 (39) 12/18 (66) 0.060

On ECMO

ECMO duration, days 8.3 (3.6-16.1) 7.5 (5.4-13.6) 0.898

ECMO weaning, n (%) 20 (61) 14 (78) 0.214

iNO on ECMO, n (%) 13 (39) 7 (39) 0.972

PP on ECMO, n (%) 4 (12) 0 (0) 0.124

LOS ICU, days 18.7 (11.5-36.7) 28.7 (19.5-46.1) 0.156
