# Supplementary material for: Retrospective analysis of factors associated with outcome in veno-venous extra-corporeal membrane oxygenation
Source: BMC Pulm Med. 2023 Aug 16;23:301. doi: 10.1186/s12890-023-02591-5 (PMC10429070; doi:10.1186/s12890-023-02591-5)
Supplement: Supplementary file 4 — Additional file 4. Arterial blood gas data. [file 12890_2023_2591_MOESM4_ESM.docx]

Additional File 4. Arterial blood gas data

Variable All cohort (N = 51) Dead (N=26) Alive (N = 25) p value

*Pre-ECMO*

FiO_2_ 1.00 (0.90-1.00) 1.00 (0.99-1.00) 1.00 (0.73-1.00) 0.113

pH 7.24 (7.11-7.33) 7.24 (7.14-7.32) 7.24 (7.07-7.36) 0.826

PaCO_2_ 62 (53-79) 62 (53-77) 61 (51-91) 0.944

PaO_2_ 62 (51-82) 59 (51-79) 69 (51-87) 0.400

P/FO_2_ 65 (52-95) 61 (51-84) 83 (54-118) 0.156

SaO_2_ 89.4 (82.9-94.2) 89.1 (82.0-91.9) 91.3 (83.7-95.4) 0.234

Lactate 1.9 (1.1-4.0) 1.9 (1.0-4.3) 2.0 (1.1-3.9) 0.888

*3h on ECMO*

FiO_2_ 0.60 (0.41-0.90) 0.60 (0.49-1.00) 0.55 (0.40-0.75) 0.254

pHa 7.37 (7.30-7.47) 7.40 (7.31-7.47) 7.35 (7.29-7.43) 0.299

PaCO_2_ 39 (34-43) 37 (33-43) 39 (35-44) 0.638

PaO_2_ 74 (66-91) 71 (61-93) 80 (69-91) 0.224

P/FO_2_ 125 (93-193) 117 (92-163) 141 (95-216) 0.155

SaO_2_ 95.3 (92.4-97.5) 95.1 (92.4-97.7) 95.3 (93.2-97.4) 0.806

Lactate 3.1 (1.7-5.7) 3.2 (1.7-6.1) 3.1 (1.4-4.6) 0.559

ARDS cohort (N=33) ARDS-Dead (N=20) ARDS-Alive (N=13) p value

*Pre-ECMO*

FiO_2_ 1.00 (0.95-1.00) 1.00 (0.99-1.00) 1.00 (0.85-1.00) 0.287

pHa 7.23 (7.11-7.30) 7.23 (7.11-7.29) 7.24 (7.11-7.31) 0.927

PaCO_2_ 62 (52-77) 62 (53-81) 59 (48-62) 0.210

PaO_2_ 59 (51-75) 60 (51-79) 56 (50-71) 0.367

P/FO_2_ 62 (52-87) 62 (51-87) 65 (52-87) 0.941

SaO_2_ 89.1 (82.0-92.2) 89.2 (82.9-92.3) 88.7 (76.6-92.7) 0.606

Lactate 2.3 (1.1-4.0) 2.3 (0.9-4.1) 2.3 (1.4-3.9) 0.768

*3h on ECMO*

FiO_2_ 0.60 (0.40-1.00) 0.70 (0.46-1.00) 0.41 (0.40-0.85) 0.263

pHa 7.36 (7.27-7.47) 7.40 (7.27-7.49) 7.33 (7.27-7.43) 0.311

PaCO_2_ 38 (33-42) 37 (33-44) 39 (34-41) 0.797

PaO_2_ 69 (59-86) 69 (58-87) 69 (59-87) 0.839

P/F O_2_ 116 (91-183) 114 (91-142) 145 (84-216) 0.338

SaO_2_ 94.2 (91.6-96.5) 94.2 (91.4-97.2) 95.1 (91.4-96.1) 0.712

Lactate 3.1 (1.7-5.9) 3.0 (1.7-6.0) 3.1 (1.8-4.6) 0.868
