# Supplementary material for: Retrospective analysis of factors associated with outcome in veno-venous extra-corporeal membrane oxygenation
Source: BMC Pulm Med. 2023 Aug 16;23:301. doi: 10.1186/s12890-023-02591-5 (PMC10429070; doi:10.1186/s12890-023-02591-5)
Supplement: Supplementary file 5 — Additional file 5. Arterial blood gas data in ARDS and non-ARDS patients. [file 12890_2023_2591_MOESM5_ESM.docx]

Additional File 5. Arterial blood gas data in ARDS and non-ARDS patients

Variable All (n=51) ARDS (n=33) non-ARDS (n=18) p value

*Pre-ECMO*

FiO_2_ 1.00 (0.90-1.00) 1.00 (0.95-1.00) 1.00 (0.51-1.00) 0.306

pHa 7.24 (7.11-7.33) 7.23 (7.11-7.30) 7.30 (7.07-7.39) 0.311

PaCO_2_ 62 (53-79) 62 (52-77) 62 (53-95) 0.587

PaO_2_ 62 (51-82) 59 (51-75) 77 (51-100) 0.103

P/FO_2_ 65 (52-95) 62 (52-87) 93 (51-171) 0.086

SaO_2_ 89.4 (82.9-94.2) 89.1 (82.0-92.2) 93.6 (83.9-98.3) 0.072

Lactate 1.9 (1.1-4.0) 2.3 (1.1-4.0) 1.7 (0.9-4.7) 0.725

*3h on ECMO*

FiO_2_ 0.60 (0.41-0.90) 0.60 (0.40-1.00) 0.60 (0.50-0.80) 0.960

pHa 7.37 (7.30-7.47) 7.36 (7.27-7.47) 7.39 (7.34-7.47) 0.567

PaCO_2_ 39 (34-43) 38 (33-42) 39 (36-46) 0.287

PaO_2_ 74 (66-91) 69 (59-86) 83 (73-111) 0.003*

P/FO_2_ 125 (93-193) 116 (91-183) 142 (122-220) 0.139

SaO_2_ 95.3 (92.4-97.5) 94.2 (91.6-96.5) 97.3 (95.3-99.0) 0.004*

Lactate 3.1 (1.7-5.7) 3.1 (1.7-5.9) 3.3 (1.1-5.2) 0.851
