# Supplementary material for: Retrospective analysis of factors associated with outcome in veno-venous extra-corporeal membrane oxygenation
Source: BMC Pulm Med. 2023 Aug 16;23:301. doi: 10.1186/s12890-023-02591-5 (PMC10429070; doi:10.1186/s12890-023-02591-5)
Supplement: Supplementary file 6 — Additional file 6. Ventilator settings and monitoring variables in ARDS and non-ARDS patients. [file 12890_2023_2591_MOESM6_ESM.docx]

Additional File 6. Ventilator settings and monitoring variables in ARDS and non-ARDS patients

Variable ARDS (N=33) non-ARDS (N=18) p value

*Pre-ECMO*

Vt (ml/kg PBW) 5.8 (5.0-7.1) 4.8 (4.1-5.7) 0.016*

Pplat (cm H_2_O) 29.0 (26.0-34.0) 29.0 (21.0-35.0) 0.897

DP (cm H_2_O) 18.0 (16.0-25.0) 26.0 (13.0-29.0) 0.257

C_RS_ (ml/cm H_2_O) 21.4 (14.2-28.9) 13.4 (9.9-23.2) 0.074

RR (min^-1^) 24.0 (20.0-28.0) 24.5 (20.0-26.5) 0.801

PEEP (cm H_2_O) 8.0 (5.0-14.0) 5.0 (2.0-8.5) 0.012*

Power (J/min) 25.6 (17.4-30.9) 17.7 (11.9-25.6) 0.049*

*24h on ECMO*

Vt (ml/kg PBW) 3.6 (2.5-4.5) 3.1 (2.4-4.0) 0.324

Pplat (cm H_2_O) 23.0 (20.3-27.8) 21.0 (19.0-25.8) 0.124

DP (cm H_2_O) 14.0 (11.0-18.0) 17.0 (12.0-20.8) 0.293

C_RS_ (ml/cm H_2_O) 18.0 (10.5-25.2) 13.9 (8.4-15.9) 0.108

RR (min^-1^) 14.0 (10.5-15.0) 15.0 (8.0-15.3) 0.682

PEEP (cm H_2_O) 9.5 (6.3-12.0) 5.0 (2.5-7.5) <0.001*

Power (J/min) 6.1 (4.2-8.4) 4.0 (2.9-5.7) 0.011*

*48h on ECMO*

Vt (ml/kg PBW) 4.3 (3.2-5.5) 3.9 (2.4-5.2) 0.562

Pplat (cm H_2_O) 23.5 (21.0-26.8) 21.0 (17.0-27.2) 0.221

DP (cm H_2_O) 14.0 (12.0-16.0) 14.0 (12.0-24.5) 0.732

C_RS_ (ml/cm H_2_O) 19.1 (11.9-28.5) 16.3 (8.0-23.7) 0.227

RR (min^-1^) 15.0 (12.0-16.0) 13.5 (8.0-16.3) 0.259

PEEP (cm H_2_O) 8.0 (7.0-12.0) 5.0 (3.0-8.5) 0.002*

Power (J/min) 7.7 (5.3-11.6) 6.0 (3.3-7.0) 0.023*
