# Supplementary material for: Retrospective analysis of factors associated with outcome in veno-venous extra-corporeal membrane oxygenation
Source: BMC Pulm Med. 2023 Aug 16;23:301. doi: 10.1186/s12890-023-02591-5 (PMC10429070; doi:10.1186/s12890-023-02591-5)
Supplement: Supplementary file 7 — Additional file 7. Ventilatory strategies during the 8 years of the study in the whole population. Patients were grouped in 4 periods of 2 y each. Differences across years were analyzed by ANOVA. [file 12890_2023_2591_MOESM7_ESM.docx]

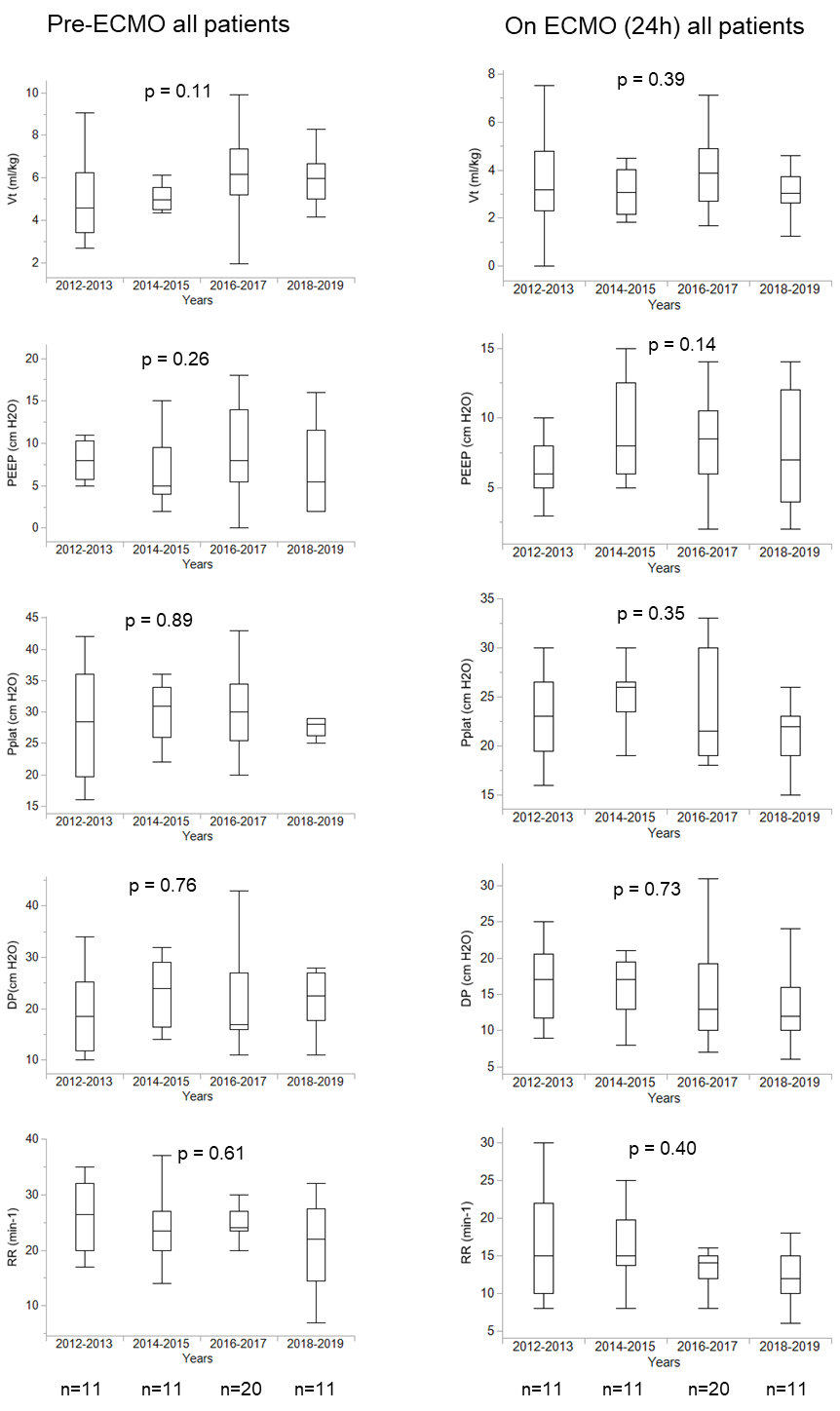
Additional File 7. Ventilatory strategies during the 8 years of the study in the whole population. Patients were grouped in 4 periods of 2 y each. Differences across years were analyzed by ANOVA.
