# Supplementary material for: Retrospective analysis of factors associated with outcome in veno-venous extra-corporeal membrane oxygenation
Source: BMC Pulm Med. 2023 Aug 16;23:301. doi: 10.1186/s12890-023-02591-5 (PMC10429070; doi:10.1186/s12890-023-02591-5)
Supplement: Supplementary file 9 — Additional file 9. Variables associated with ICU mortality: univariate analysis. [file 12890_2023_2591_MOESM9_ESM.docx]

Additional File 9. Variables associated with ICU mortality: univariate analysis

All patients ARDS patients

Univariate OR [95% CI] p value OR [95% CI] p value

ARDS vs non-ARDS 3.07 [0.92-10.25] 0.060

Days MV before ECMO 1.04 [0.95-1.14] 0.355 1.40 [1.09-2.04] 0.003*

SAPS 2 1.09 [0.95-1.24] 0.431 1.00 [0.96-1.04] 0.807

SOFA first 24h 1.10 [0.96-1.27] 0.229 1.06 [0.88-1.31] 0.537

P/FO_2_ pre-ECMO 0.99 [0.98-1.00] 0.073 1.00 [0.97-1.03] 0.955

Vt pre-ECMO 0.77 [0.53-1.13] 0.163 0.75 [0.45-1.15] 0.188

Vt 24h on ECMO 0.93 [0.63-1.37] 0.700 0.95 [0.59-1.54] 0.843

Vt 48h on ECMO 0.72 [0.49-1.08] 0.110 0.82 [0.50-1.30] 0.410

Pplat pre-ECMO 1.02 [0.92-1.12] 0.722 0.98 [0.84-1.12] 0.721

Pplat 24h on ECMO 1.18 [1.02-1.36] 0.015* 1.13 [0.96-1.37] 0.143

Pplat 48h on ECMO 1.16 [1.01-1.32] 0.014* 1.08 [0.92-1.26] 0.346

DP pre-ECMO 1.04 [0.96-1.13] 0.319 1.14 [0.99-1.35] 0.063

DP 24h on ECMO 1.14 [1.01-1.28] 0.021* 1.19 [1.02-1.45] 0.023*

DP 48h on ECMO 1.14 [1.03-1.33] 0.012* 1.16 [0.99-1.47] 0.075

C_RS_ pre-ECMO 0.96 [0.90-1.02] 0.137 0.94 [0.86-1.01] 0.082

C_RS_ 24h on ECMO 0.92 [0.85-0.99] 0.019* 0.91 [0.83-0.99] 0.027*

C_RS_ 48h on ECMO 0.94 [0.88-0.99] 0.024* 0.95 [0.88-1.01] 0.116

RR pre-ECMO 1.06 [0.96-1.17] 0.213 1.11 [0.98-1.30] 0.103

RR 24h on ECMO 1.05 [0.93-1.18] 0.389 1.19 [0.96-1.56] 0.128

RR 48h on ECMO 0.42 [0.10-1.67] 0.402 1.13 [0.94-1.44] 0.214

PEEP pre-ECMO 0.93 [0.82-1.06] 0.274 0.78 [0.62-0.93] 0.005*

PEEP 24h on ECMO 0.96 [0.82-1.13] 0.629 0.83 [0.65-1.04] 0.101

PEEP 48h on ECMO 0.96 [0.81-1.13] 0.640 0.87 [0.66-1.10] 0.250

Power pre-ECMO 1.05 [0.99-1.13] 0.109 1.07 [0.99-1.18] 0.102

Power 24h on ECMO 0.97 [0.80-1.16] 0.709 0.96 [0.76-1.20] 0.727

Power 48h on ECMO 1.01 [0.86-1.19] 0.858 1.03 [0.84-1.26] 0.764
